# Supplementary material for: Divergent recovery trajectories of intertidal and subtidal coral communities highlight habitat-specific recovery dynamics following bleaching in an extreme macrotidal reef environment
Source: PeerJ. 2023 Sep 15;11:e15987. doi: 10.7717/peerj.15987 (PMC10506583; doi:10.7717/peerj.15987)
Supplement: Supplemental Information 1 [file peerj-11-15987-s001.pdf]

Divergent recovery trajectories of intertidal and subtidal coral communities highlight habitat-specific recovery dynamics following bleaching in an extreme macrotidal reef environment

Elias Speelman[1], Michael Parger[2], Verena Schoepf[1,2]

[1] Department of Freshwater and Marine Ecology, Institute for Biodiversity and Ecosystem Dynamics, University of Amsterdam, Science Park 904, 1098 XH Amsterdam, The Netherlands [2] UWA Ocean Institute, The University of Western Australia, 35 Stirling Highway, Perth WA 6009, Australia

Corresponding Author:
Verena Schoepf
Science Park 904, 1098 XH Amsterdam, The Netherlands
Email address: v.schoepf@uva.nl

Supplemental Information

Supplemental Tables

Supplemental Table S1: Species to genera life history strategy (LHS) conversion table. LHS was determined for all genera recorded in the surveys as per Darling et al. (2012) for Indo-Pacific species occurring in the Kimberley Coast, north-west Australia (ER091) (see Methods). C = competitive, G = generalist, S = stress-tolerant, W = weedy.

**Genera where all species have the same LHS**	**Genera where LHS was determined based on species ID**	**Genera excluded due to uncertainty regarding LHS**	**Genera excluded since they were not mentioned in Darling et al. (2012)**
Acropora (C)	*Pocillopora*[2] *(W)*	*Goniastrea (W or S)*	*Coeloseris*
Astreopora (S)		*Montipora (C, G or S)*	*Ctenactis*
Caulastrea (S)		*Pavona (G or S)*	*Euphyllia*
Dispsastrea = Favia (S)		*Porites (S, C or W)*	*Goniopora*
Favites (S)		*Turbinaria (G or C)*	*Herpolitha*
Fungia (S)			*Leptoseris*
Galaxea (S)			*Millepora*
Lobophyllia (S)			*Trachyphyllia*
Montastrea[1] *(S)*			
Platygyra (S)			
Seriatopora (W)			
Stylophora (W)			

[1] the two *Montastrea* species listed in Darling et al. (2012) now belong to different genera: *Montastrea curta* = *Astrea curta*; *Montastrea valenciennesi* = *Phymastrea valenciennesi* (Veron et al., 2023).

[2] the dominant species in our surveys was identified to be *Pocillopora acuta* based on macro-morphology (Fig. S2) which used to be synonymous with *Pocillopora damicornis.* Since the latter was assigned a weedy LHS by Darling et al. (2012), we used this LHS for the genus *Pocillopora* in our analysis – see Methods for more details.

Supplemental Table S2: Statistical output from the PERMDISP analysis for benthic cover, coral community composition, coral morphology, life history strategy and corallite integration index score (CIIS). Separate analyses were done (1) using all four time points and (2) comparing the 3.5 year recovery time point (Oct 2019) to the pre-bleaching time point only (Jan 2016). Statistically significant p-values (<0.05) are highlighted in bold. N.Perm = number of permutations.

		Df	Sum Sq	Mean Sq	F	N.Perm	Pr(>F)
**Benthic cover** (4 time points)	Groups	7	0.10318	0.0147397	2.1683	999	0.050
	Residuals	41	0.27871	0.0067979			
**Benthic cover** (2 time points)	Groups	3	0.000964	0.0003214	0.0571	999	0.988
	Residuals	21	0.118311	0.0056339			
**Coral comm. composition** (4 time points)	Groups	7	0.18441	0.026344	1.9298	999	0.089
	Residuals	41	0.55969	0.013651			
**Coral comm. composition** (2 time points)	Groups	3	0.16351	0.054504	3.2912	999	**0.037**
	Residuals	21	0.34778	0.016561			
**Coral morph.** (4 time points)	Groups	7	0.26667	0.038095	1.8936	999	0.096
	Residuals	41	0.82485	0.020118			
**Coral morph.** (2 time points)	Groups	3	0.24679	0.082264	6.2316	999	**0.003**
	Residuals	21	0.27722	0.013201			
**Life history strategy** (4 time points)	Groups	7	0.26175	0.037392	1.5074	999	0.181
	Residuals	41	1.01706	0.024806			
**Life history strategy** (2 time points)	Groups	3	0.09279	0.03093	1.9391	999	0.162
	Residuals	21	0.33496	0.01595			
**CIIS** (4 time points)	Groups	7	0.22066	0.031523	2.5844	999	**0.023**
	Residuals	41	0.50008	0.012197			
**CIIS** (2 time points)	Groups	3	0.018719	0.0062395	0.8272	999	0.473
	Residuals	21	0.158395	0.0075426			

Supplemental Table S3: Results from PERMANOVA analyses testing for the effect of time (3.5 year recovery time point [Oct 2019], pre-bleaching time point [Jan 2016]) and zone (intertidal vs subtidal) on benthic cover, coral community composition, coral morphology, life history strategy and corallite integration index score (CIIS).Statistically significant p-values (<0.05) are highlighted in bold.

	Factor	Df	SumsOfSqs	MeanSqs	F.Model	R2	Pr(>F)
**Benthic cover** (2 time points)	Time	1	0.04660	0.046596	3.7678	0.11672	**0.042**
	Zone	1	0.00863	0.008627	0.6976	0.02161	0.528
	Time:Zone	1	0.08427	0.084270	6.8143	0.21110	**0.003**
	Residuals	21	0.25970	0.012367		0.65056	
	Total	24	0.39919			1.00000	
**Coral community composition** (2 time points)	Time	1	0.30406	0.30406	4.5258	0.12113	**0.009**
	Zone	1	0.29901	0.29901	4.4505	0.11912	**0.009**
	Time:Zone	1	0.49615	0.49615	7.3850	0.19766	**0.001**
	Residuals	21	1.41087	0.06718		0.56208	
	Total	24	2.51008			1.00000	
**Coral morphology** (2 time points)	Time	1	0.02126	0.021264	0.4508	0.01723	0.622
	Zone	1	0.20644	0.206440	4.3766	0.16726	**0.025**
	Time:Zone	1	0.01598	0.015979	0.3388	0.01295	0.705
	Residuals	21	0.99056	0.047169		0.80256	
	Total	24	1.23424			1.00000	
**Life history strategy** (2 time points)	Time	1	0.23874	0.23874	12.891	0.18819	**0.001**
	Zone	1	0.35475	0.35475	19.155	0.27963	**0.001**
	Time:Zone	1	0.28620	0.28620	15.454	0.22560	**0.001**
	Residuals	21	0.38892	0.01852		0.30657	
	Total	24	1.26861			1.0000	
**Corallite integration index score** (2 time points)	Time	1	0.17504	0.17504	14.970	0.20022	**0.002**
	Zone	1	0.33095	0.33095	28.304	0.37857	**0.001**
	Time:Zone	1	0.12268	0.12268	10.492	0.14033	**0.005**
	Residuals	21	0.24555	0.01169		0.28087	
	Total	24	0.87423			1.00000	

Supplemental Figures

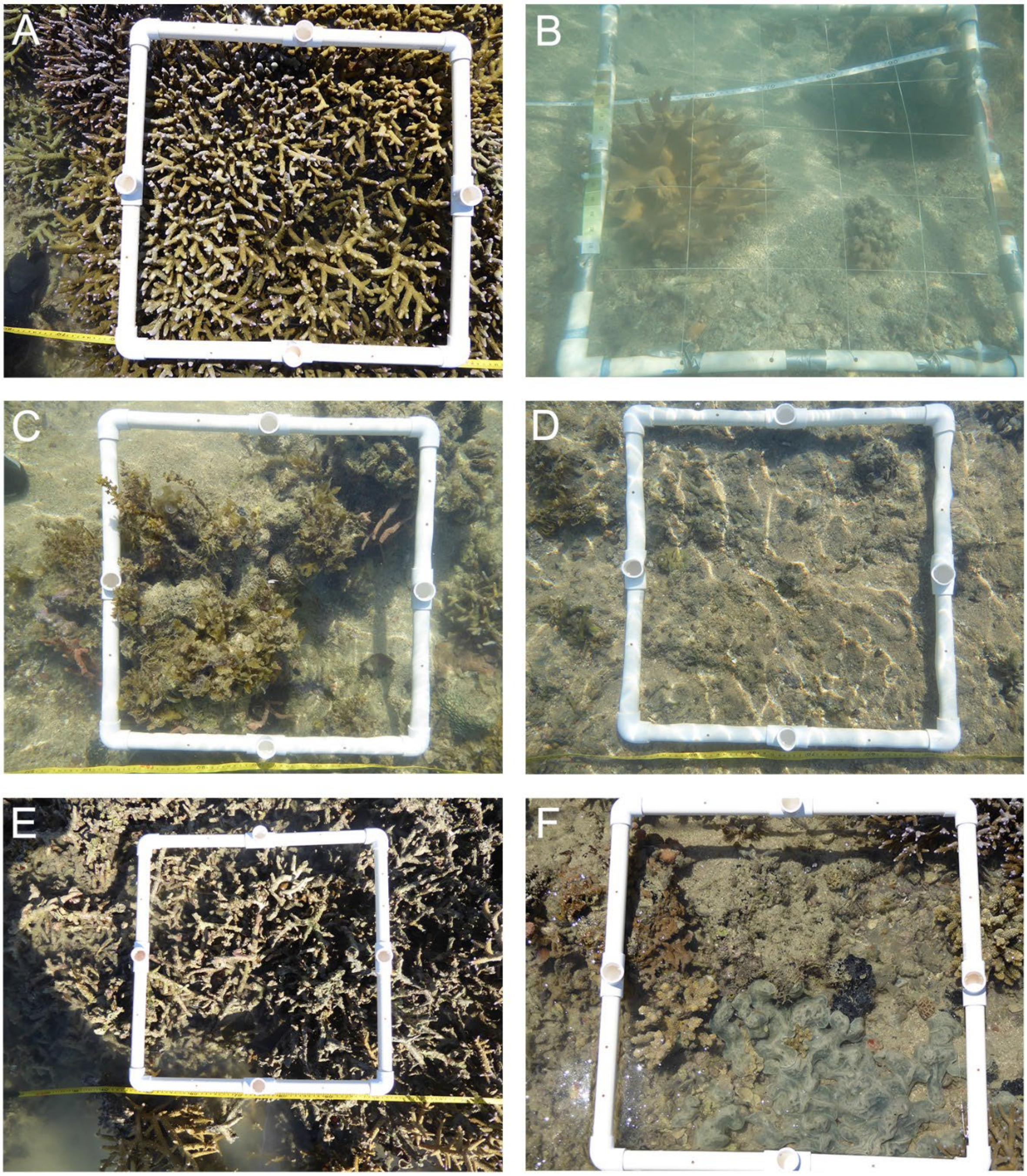

Supplemental Figure S1. Benthic cover categories: photo-quadrats showing examples of (A) live hard coral, (B) soft coral, (C) algae, (D) substrate, (E) recently deceased coral and (F) “unknown/other” (anything that did not fit in the aforementioned categories or was unclear). The category “substrate” was used for all types of abiotic benthic cover such as rubble, sand or rocks. Corals were categorized as recently deceased if they were presumed to have died in the last few months, as indicated by a general coral shape still being recognizable. If a coral had already disintegrated into rubble, it was considered dead for a longer period of time and thus categorized as substrate.

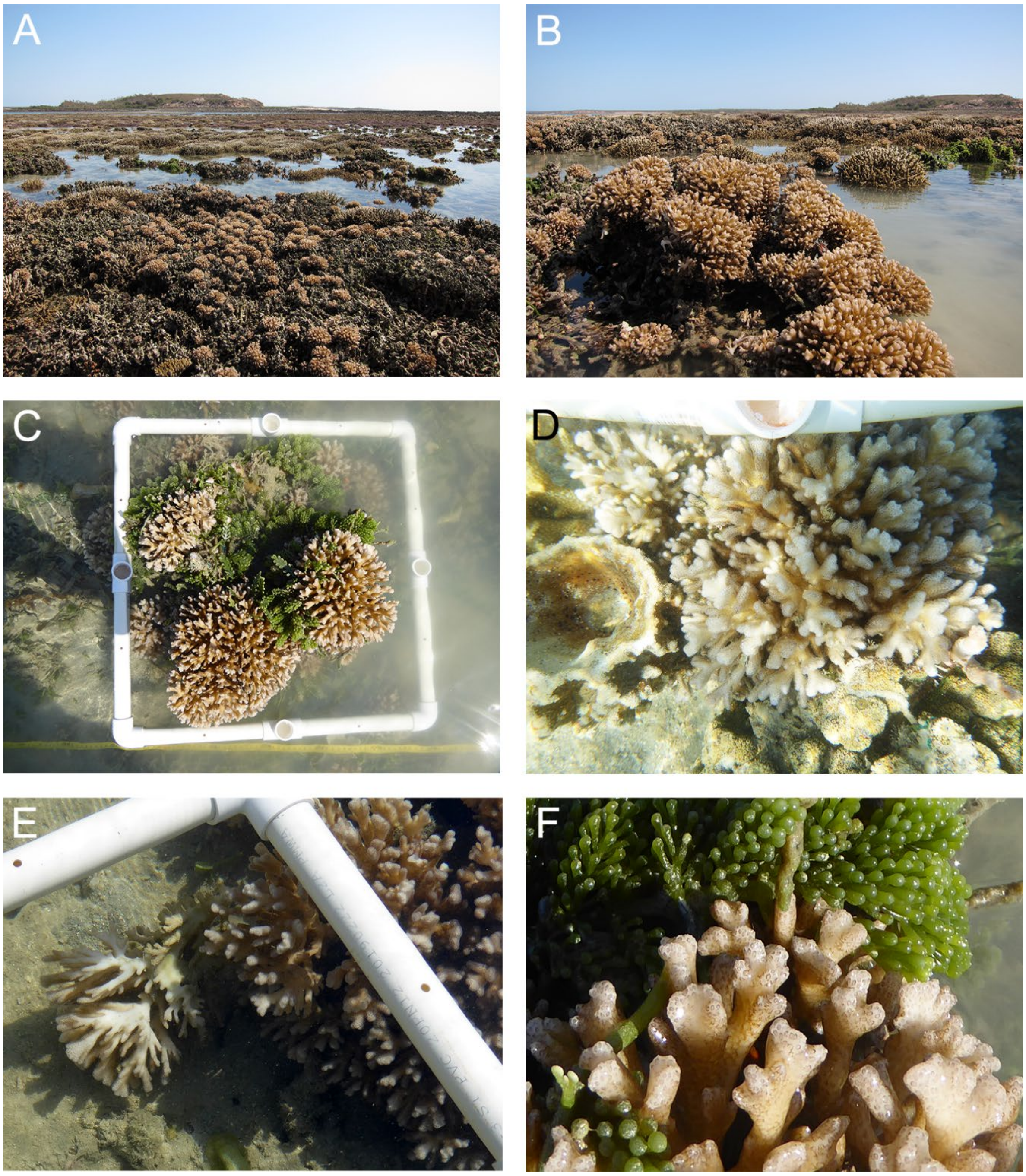

Supplemental Figure S2. (A-B) High abundance of *Pocillopora* colonies, often growing on dead *Acropora* corals, in the subtidal zone in October 2019. (C-D) *Pocillopora* colonies recorded in the surveys in October 2019. (E-F) Close-up photos of *Pocillopora* colonies. The species was identified to be *P. acuta* based on macro-morphology (see Methods) (Schmidt-Roach et al., 2014).

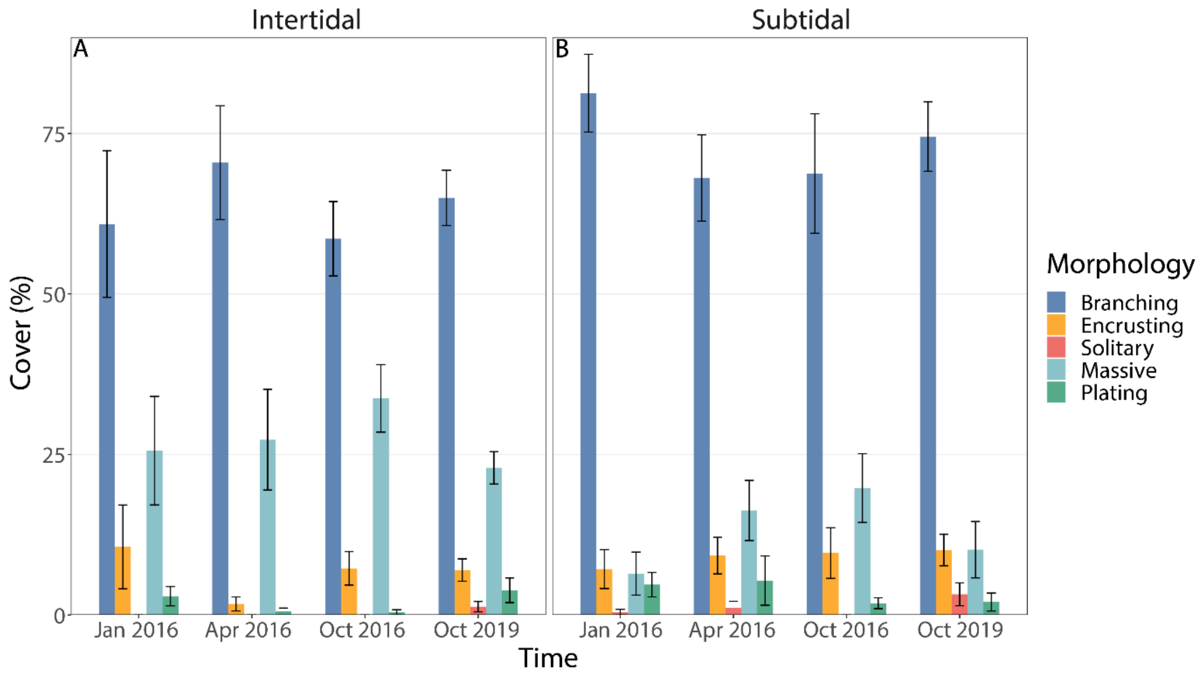

Supplemental Figure S3. Percentage cover of coral genera with a certain morphology across all four time points in the (A) intertidal and (B) subtidal zone. Shown is mean ± 1SE.

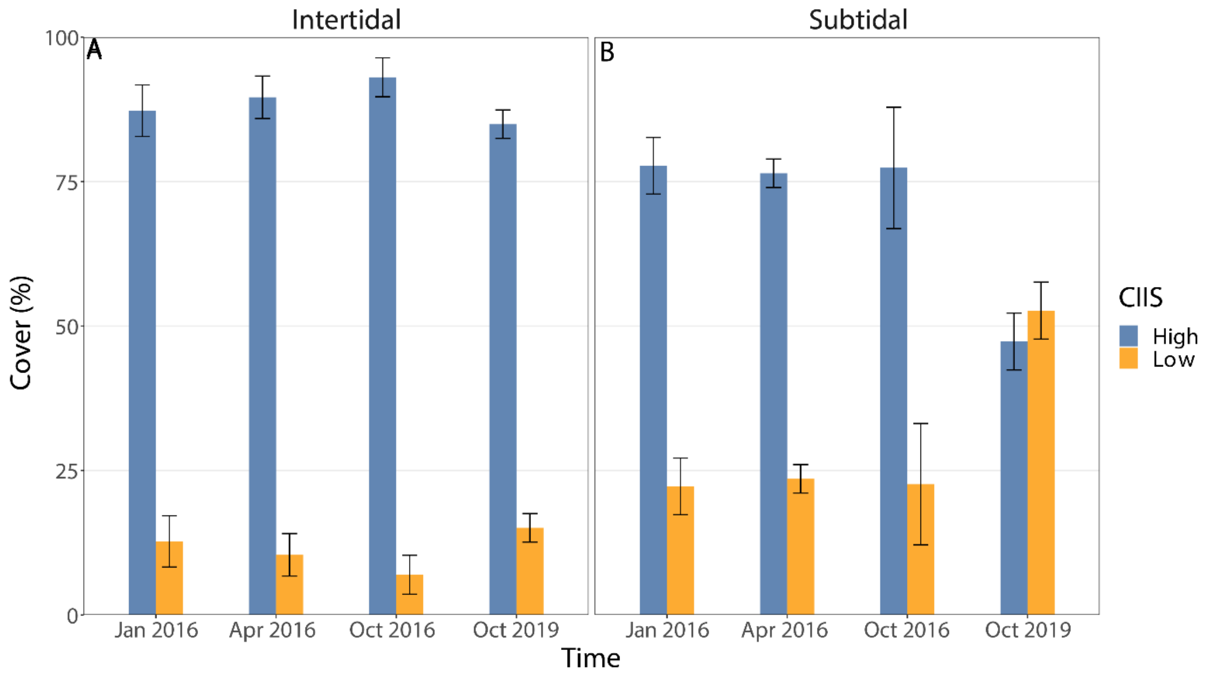

Supplemental Figure S4. Percentage cover of coral genera with high or low corallite integration index score (CIIS) across all four time points in the (A) intertidal and (B) subtidal zone. Shown is mean ± 1SE.

References

Darling ES, Alvarez-Filip L, Oliver TA, McClanahan TR, Côté IM. 2012. Evaluating life-history strategies of reef corals from species traits. *Ecology Letters* 15:1378–1386. DOI: https://doi.org/10.1111/j.1461-0248.2012.01861.x.

Schmidt-Roach S, Miller KJ, Lundgren P, Andreakis N. 2014. With eyes wide open: a revision of species within and closely related to the Pocillopora damicornis species complex (Scleractinia; Pocilloporidae) using morphology and genetics. *Zoological Journal of the Linnean Society* 170:1–33. DOI: https://doi.org/10.1111/zoj.12092.

Veron JEN, Stafford-Smith MG, Turak E, DeVantier LM. 2023.Corals of the World, Accessed January 2023, version 0.01. *Available at http://coralsoftheworld.org*
